# Investigation into the utility of ultra-high-resolution mode on dynamic-ventilation computed tomography using sponge phantoms

**DOI:** 10.1038/s41598-025-30102-5

**Published:** 2025-12-11

**Authors:** Ryo Uemura, Yukihiro Nagatani, Jun Matsubayashi, Akitoshi Inoue, Kyohei Iwai, Kenichi Kamiya, Kentaro Doi, Akira Sato, Hodaka Numasaki, Kazuaki Nakane, Masahiro Yanagawa, Noriyuki Tomiyama, Yoshiyuki Watanabe

**Affiliations:** 1 Department of Radiology, Shiga University of Medical Science, Seta Tsukinowa-cho, Otsu, Shiga 520-2192 Japan; 2 Center for Clinical Research and Advanced Medicine, Shiga University of Medical Science, Seta Tsukinowa-cho, Otsu, Shiga 520-2192 Japan; 3 Division of Cardiovascular Surgery, Department of Surgery, Shiga University of Medical Science, Seta Tsukinowa-cho, Otsu, Shiga 520-2192 Japan; 4 Department of Medical Physics and Engineering, The University of Osaka Graduate School of Medicine, 2-2 Yamadaoka, Suita, Osaka 565-0871 Japan; 5 Artificial Intelligence Center for Health and Biomedical Research, National Institutes of Biomedical Innovation, Health and Nutrition, 3-17 Senrioka-shin-machi, Settsu-shi, Osaka, 566-0002 Japan; 6 Department of Radiology, The University of Osaka Graduate School of Medicine, 2-2 Yamadaoka, Suita, Osaka 565-0871 Japan

**Keywords:** Computed tomography, Medical imaging, Three-dimensional imaging, Respiratory tract diseases

## Abstract

Ultra-high-resolution (UHR) computed tomography (CT) provides more detailed information on peripheral lung structures than normal-resolution (NR) CT for static images. However, its potential advantage in four-dimensional dynamic-ventilation CT (DVCT) has not been investigated. This experimental study used sponge phantoms and a compression machine to simulate respiratory changes and assess the superiority of the UHR mode on DVCT. We obtained results that demonstrated: (1) regardless of the compression percentage, the number of simulated peripheral air spaces (SPAS) detected on UHR-CT was greater than on NR-CT, as assessed using a simple binarization method. Similarly, peak *b*_*0*_ values as a representative of total SPAS count obtained by homology-based image analysis were higher on UHR-CT than on NR-CT. (2) The deviation in measured longitudinal diameter and area of the SPAS relative to the reference standard—smartphone images—was smaller on UHR-CT than on NR-CT. (3) UHR DVCT demonstrated a gradual reduction in the longitudinal diameter and area of SPAS with increasing compression percentages, consistent with smartphone images, whereas NR DVCT did not. These findings suggested that UHR mode is superior to NR mode in depicting peripheral lung structures for DVCT as well as static images.

**Supplementary Information:**

The online version contains supplementary material available at 10.1038/s41598-025-30102-5.

## Introduction

Imaging diagnosis for pulmonary diseases is primarily based on morphological evaluation modalities obtained at peak-inspiratory breath-hold, such as chest X-ray and computed tomography (CT). Previous studies using high-resolution CT, with pixel sizes of 0.5 to 2 mm, have identified more useful and specific radiological findings for numerous pulmonary diseases, including chronic obstructive pulmonary disease, interstitial lung disease, and pulmonary malignant tumors. Recently, ultra-high-resolution (UHR) CT with pixel sizes of 0.25 mm or less has been developed and made commercially available, and its clinical usefulness has been reported for the depiction of smaller structures such as peripheral bronchi^1–3^.

Pulmonary functional imaging has been recognized as more essential for the evaluation of abnormalities that conventional static imaging modalities cannot depict and for evaluating therapeutic effects. Ventilation and perfusion scintigraphy and paired inspiratory and expiratory CT are complimentarily used with additional information for regional or heterogeneous impairment in clinical practice as pulmonary functional imaging modalities^4–6^. However, information provided by these modalities are not necessarily sufficient. In contrast, several novel imaging modalities have recently shown promise for evaluating pulmonary function. These include dynamic chest radiography^7,8^, ventilation imaging with dual-energy CT or magnetic resonance imaging (MRI) using noble gases (such as xenon)^9,10^, pulmonary functional MRI without contrast agents (such as phase-resolved functional lung MRI)^11,12^, and four-dimensional (4D)-dynamic ventilation CT (DVCT).

Among these, previous studies using image data from DVCT have highlighted new perspectives, including the usefulness of preoperative evaluation for pleural adhesion^13,14^, the association between pleural movement and smoking^15^, dynamic respiratory changes of central airways in smokers including those with COPD^16^, and strain analysis of abnormal respiratory deformation in the lungs of patients with COPD^17^. However, to the best of our knowledge, no studies have focused on peripheral lung structures using DVCT. As described above, although UHR-CT is useful for evaluating peripheral lung structures, several drawbacks should be considered. Currently, the commercially available UHR-CT has a limited scanning coverage in a cranio-caudal axis, and image noise and respiratory or cardiac motion artifacts are expected to be more prominent than in normal-resolution (NR)-CT when the UHR mode is applied to DVCT. Nonetheless, DVCT based on the UHR mode is expected to be useful for the assessment of functional impairment in peripheral lung structures. To demonstrate the feasibility of its clinical implication, a detailed experimental study using a simulated phantom is desirable as a preliminary evaluation. Sponges were used as deformable phantoms to simulate internal lung structures in previous research on respiratory-gated radiotherapy for lung tumors using 4D CT^18^. Therefore, in the present study, we conducted an experimental investigation using sponge phantoms that are inexpensive, readily available, and easy to fit into the compression device.

In this study, we developed sponge phantoms to simulate internal lung structures, scanned them during gradual compression and compared dynamic change of simulated peripheral air spaces (SPAS) between UHR-CT and NR-CT based on detailed quantitative evaluation.

## Materials and methods

**Fig. 1:**
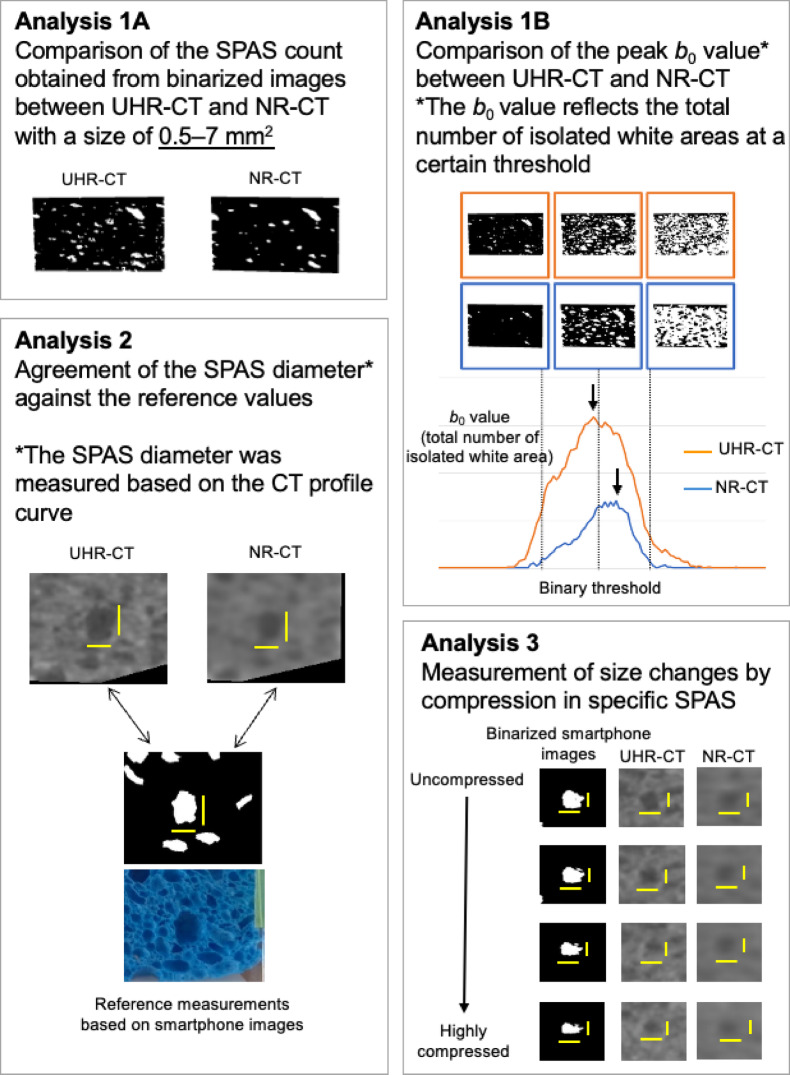
Analysis overview of the study.

The following quantitative measurements were performed in an experimental approach using a simulated sponge phantom including a lot of SPAS with different sizes under simulated expiratory conditions, to demonstrate whether the visibility of SPAS on dynamic CT obtained in UHR mode was superior to that obtained in NR mode. An overview of the analysis in this study is shown in Fig. 1. First, binarization by the function with the highest visual concordance with an original image was used to measure the number and size of SPAS (Analysis 1A). Homology analysis was used as a multi-threshold strategy to overcome the limitation of counting SPAS with simple binarization (Analysis 1B). The longitudinal and horizontal diameters of each SPAS were measured to focus on concordance of measurement on CT images against those on smartphone images as a reference (Analysis 2) and to focus on the size changes according to the compression percentage of sponge phantoms (Analysis 3).

### Sponge phantoms and the compressing device

**Fig. 2:**
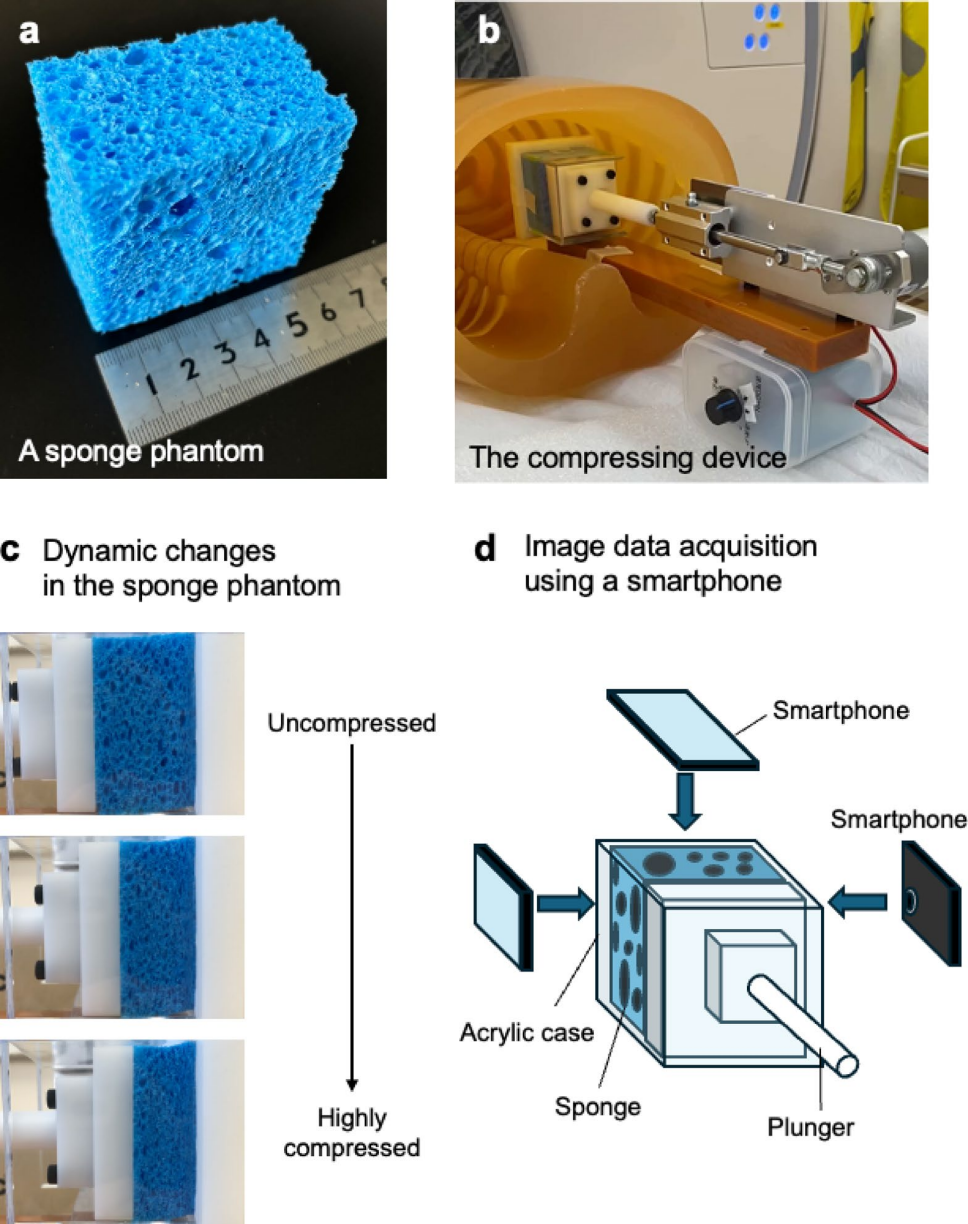
Data acquisition of dynamic images with a dedicated compressing machine. (**a**) A sponge phantom made of cellulose. (**b**) The compressing device connecting to the sponge phantom inside a transparent case was placed on the posterior thoracic wall in a chest phantom with a reproduced chest wall (N-1; Kyoto Kagaku Co., Kyoto, Japan). Surface cross-sections in sponge phantoms facing ventral and lateral thoracic wall sides of the chest phantom were defined as the upper and lateral ones. Median cross-sections were defined as the opposite side to the lateral ones. (**c**) Three intermittent images of sponge phantoms during dynamic change. (**d**) Image acquisition of the sponge phantom using a smartphone. Movie images of three surface cross sections of the sponge phantoms were obtained using a smartphone (iPhone X; Apple, Cupercino, CA, USA).

We visually compared the contrast between SPAS and the surrounding sponge matrix among several commercially available sponges made of different materials. Cellulose sponges, which showed the highest contrast, were determined to be the most appropriate for use as the simulation phantom (S-Fig. 1a, b; details on the selection of a sponge are provided in the Supplemental information). Cellulose sponges were used as phantoms that include SPAS after cutting into rectangular parallelepiped pieces of approximately 6 × 6 × 3.5 cm and immersed in diluted iodine contrast media and dried in this study (Fig. 2a).

The sponge phantoms were compressed and decompressed using a dedicated device to simulate respiratory changes in the lungs (Fig. 2b, c). In this study, the maximal compression length on the simulated sponge phantoms was fixed as 15 mm. The duration time of the compression cycle was adjustable and set at 4 s (compression velocity; approximately 7.5 mm/s) and 5 s (compression velocity; approximately 6 mm/s).

Details about the preparation of the sponge phantoms and specification of the dedicated compressing device are provided in the Supplementary information.

### Image data acquisition and reconstruction on dynamic computed tomography during simulated respiration

In general, data acquisition under rest breathing on DVCT enabled us to observe continuous dynamic changes in the lungs. In this study, the compressing device, with the sponge phantoms set inside a transparent case, was placed on the posterior thoracic wall in a commercially available chest phantom with a reproduced chest wall and mediastinum (N-1; Kyoto Kagaku Co., Kyoto, Japan) (Fig. 2b). Therefore, scan data were acquired under two kinds of simulated respiration as described above, on dynamic CT using two protocols as follows; the first was data acquisition in NR mode using a 320-row CT scanner (Aquilion ONE; Canon Medical Systems, Otawara, Tochigi, Japan) with a slice thickness of 0.5 mm, matrix of 512 × 512 and field of view (FOV) of 256 mm, and the second was data acquisition in UHR mode using a 160-row CT scanner (Precision; Canon Medical Systems, Otawara, Tochigi, Japan) with a slice thickness of 0.25 mm, matrix of 1024 × 1024 and FOV of 256 mm.

Other data acquisition and reconstruction parameters were the same for both protocols: tube current, 20 mA; tube voltage, 120 kVp; rotation time, 0.35 s; reconstruction FOV, 256 mm; image reconstruction interval, 0.35 s/frame; half reconstruction algorithm; deep-learning-based image reconstruction algorithm; and reconstruction kernel, standard kernel (FC13). FOV of images in UHR mode was cropped to 128 mm because images with a matrix size of more than 512 are unavailable on the dedicated computer workstation used for the subsequent image review and data analyses.

The effective doses calculated as the CT dose index based on the multiplication of the dose-length product values by a factor of 0.014^19^ were 0.3 mGy for NR mode and 0.5 mGy for UHR mode. The estimated motion blur was 1.31 mm/image at 4 s and 1.05 mm/image at 5 s, corresponding to the moving distance in the compressing direction.

### Image data acquisition using a smartphone as the reference standard

After dynamic CT image acquisition, the sponge phantoms set inside the transparent case of the compressing device was retrieved from the chest phantom for subsequent image data acquisition using smartphone. With 2 identical modes of simulated respiration, movie images (30 frames per second ) of 3 surfaces of the sponge phantoms, corresponding to upper, median, and lateral surfaces, were obtained using a smartphone (iPhone X; Apple, Cupertino, CA, USA), whose imaging surface was set parallel to the surfaces and whose imaging range included the entire sponge phantom (Fig. 2d). The estimated motion blur was 0.25 mm/image at 4 s and 0.19 mm/image at 5 s, corresponding to the moving distance in the compressing direction.

### Selection of evaluation image cross-sections of dynamic computed tomography

**Fig. 3:**
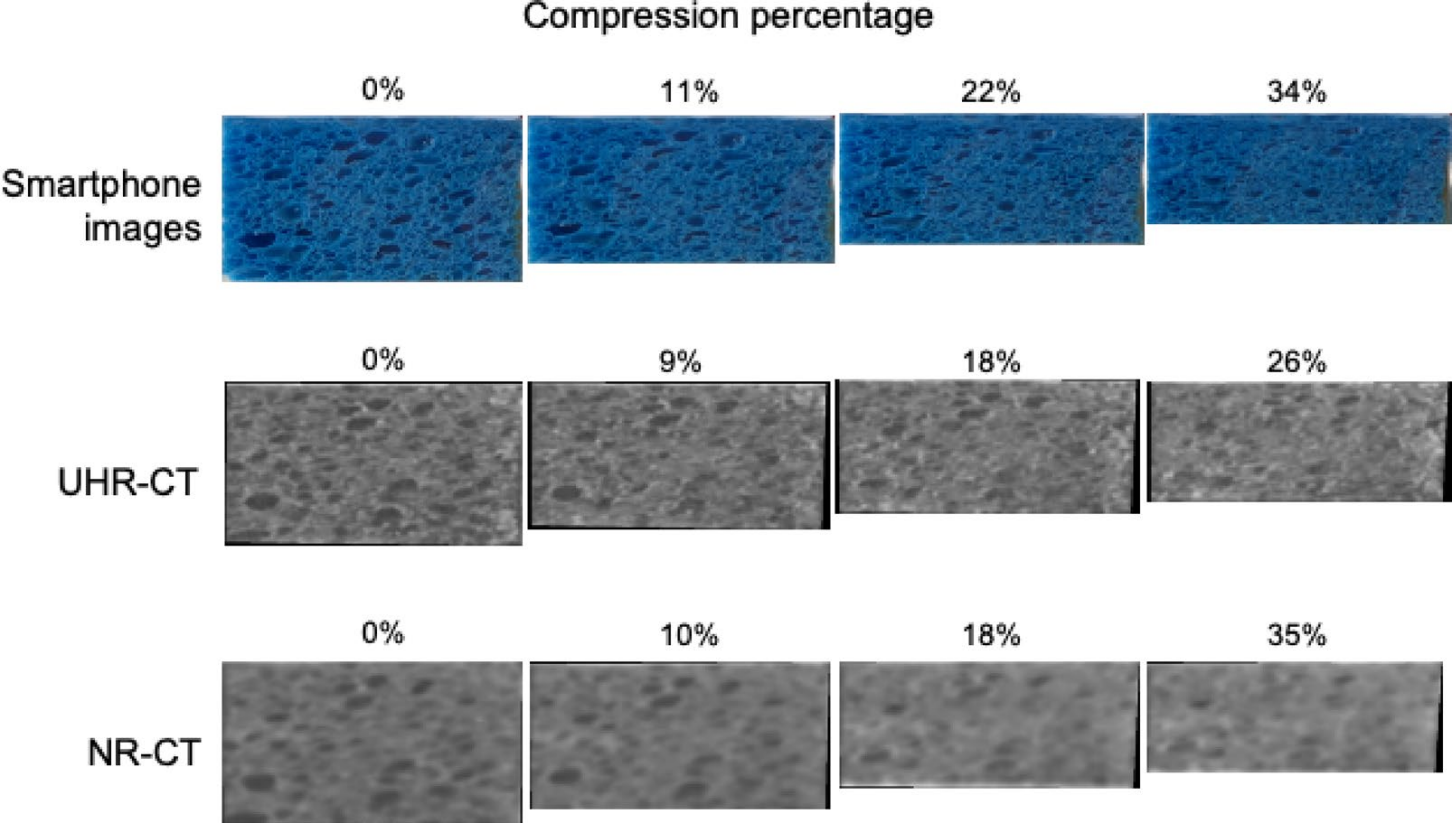
Examples of four respiratory phases simulated by different compression percentages. Smartphone, ultra-high-resolution computed tomography (CT), and normal-resolution CT images corresponding to 4 different compression percentages are demonstrated respectively in the upper, middle and lower rows. Figures over corresponding images indicate the compression percentage of the sponge phantom.

In general, the three cross-sections (upper, median, and lateral) near the surface in each sponge phantom were used as the evaluation cross-sections for quantitative assessments to compare with images of surfaces of sponge phantoms using the smartphone, which was the reference standard (S-Video). Four phases of CT images with different compression percentages of the sponge phantom were used for quantitative assessments (Fig. 3). The selection process in detail was as follows.

Image review and procedures were performed on commercially available workstation software (ZAIO station, ZAIOSOFT, Tokyo, Japan). Image data were magnified 200 times with NR mode and 400 times with UHR mode so that only the sponge phantoms were included within the observational field of view. Image data in sequential phases 7 to 9 corresponded to simulated expiration. Among these, four phases with different compression percentages including the uncompressed phase were visually selected and used for quantitative assessments, because they approximately corresponded to peak inspiration, early expiration, middle expiration, and late expiration during respiratory change of lungs from inspiration to expiration (Fig. 3). The compression percentages in UHR-CT images were not completely the same as those in corresponding NR-CT images because time phases did not necessarily coincide among CT images obtained using two different modes with different scanners. The maximal compressed phase was inappropriate for analysis because SPAS were unrecognizable on most CT images in both UHR and NR. For the six sponge phantoms, 18 cross-sections corresponding to the three surfaces captured in the smartphone movies were intended for analysis. Four cross-sections were excluded because of visual inconsistency in the distribution and configurations of SPAS between CT images and the smartphone images, probably due to distortion or bending of the surfaces of the sponge phantoms with compression.

Finally, 224 CT images in total were obtained from six sponge phantoms: 14 cross-sections ×4 phases ×2 data acquisition protocols ×2 compression cycle durations. The compression percentage of the sponge phantom in each image was calculated based on the ratio of the length of the sponge phantom along with the moving direction of the plunger of the compressing device at each compressed phase to that at the uncompressed phase. The mean CT density values (range) of the 14 cross-sections at the uncompressed phase in UHR-CT and NR-CT were − 775.8 (− 795.9 to − 757.0) and − 782.4 (− 816.5 to − 729.9) HU, respectively.

### Analysis 1A: Comparison of SPAS counts obtained from binarized CT images between UHR and NR-CT images

The following procedures were performed using Image J software, Version 2.3.0/1.53f (Rasband W.S., Image J, U.S. National Institute of Health, Bethesda, MD, USA, http://rsb.info.nih.gov/ij/, 1997–2008) on a personal computer. After automatic conversion of an original CT image into 16 predefined plural binarized ones, those were Default, Huang, Huang2, Intermodes, IsoData, Li, MaxEntropy, Mean, MinError(I), Minimum, Moments, Otsu, Percentile, RenyiEntropy, Shanbhag, Triangle, and Yen using an automatic threshold function (auto threshold), the Moments function was determined to be the most appropriate function, as it provided the highest visual concordance—with more accurate SPAS distribution and morphology compared with the other functions—closely comparable to the original image displayed with a window width of –1500 HU and a window level of 650 HU. The Moments function applies moment-preserving thresholding, a deterministic binarization method that selects the threshold so that the statistical moments of the original image histogram are preserved in the binarized image^20^ (S-Fig. 2). The number and the size of SPAS on binarized images using the Moments function were measured using the function for automatic counting of particles (Analyze Particles function) (S-Fig. 3). The Analyze Particles function of ImageJ was applied to the binary images generated after thresholding. It automatically detects discrete particles and reports their count and area, with size thresholds set to exclude irrelevant objects (e.g., noise or very small artifacts). SPAS smaller than 7 mm^2^ (approximately 3 mm in diameter) were counted, because our aim was to evaluate peripheral structures such as segmental bronchi less than 3 mm in diameter using DVCT in UHR mode. Conversely, SPAS smaller than 0.5 mm^2^ (approximately 0.8 mm in diameter) were excluded, because a considerable number of image noise artifacts might otherwise be misidentified as SPAS on binarized UHR-CT images. There is a trade-off between spatial resolution and image noise even if deep-learning-based image reconstruction with noise reduction is applied. Actually, for SPAS smaller than 0.5mm^2^, the number of SPAS detected on UHR-CT was about 8 times that on NR-CT and some noise was visually confirmed.

The number of SPAS with a size of 0.5–7 mm^2^, as counted on each binarized image, was compared between NR and UHR-CT images using a linear mixed model. Independent variables of this model were the CT mode (NR or UHR), position of the cross-section (upper, median, or lateral surface of a sponge phantom), compression cycle (4 s or 5 s), compression percentage (as a continuous variable), and interaction between the CT mode and compression percentage. The model incorporated the correlations for all SPAS counts obtained from the same cross-section of a sponge phantom. The covariance structure for the correlations was compound symmetry. Empirical variance was employed for calculating the test statistic and 95% confidence intervals.

### Analysis 1B: Comparison of the peak b_0_ value of homology representing total count between UHR and NR-CT images

Simple binarization processing can be expected to represent the number of SPAS while the results depend on the threshold value corresponding to a single display condition assumed appropriate, which is a so-called lung field window applied to the processing. This process is judged as feasible; however, a single display condition does not necessarily cover the most appropriate observation condition. Therefore, to enhance the results obtained based on simple binarization, the *b*_*0*_ value was also quantified as a representative of the total count of SPAS using homology, which was reported to be useful based on multi-threshold strategies; homology-based image analysis can capture the geometric features of images by numerical calculation. Nakane et al*.* proposed a method that quantifies the contact of nuclei in the pathological image based on homology^21^.

Homology-based image analysis provides the Betti number, which has two dimensions composed of *b*_*0*_ and *b*_*1*_. The values of *b*_*0*_ and *b*_*1*_ indicate the number of isolated pixels and the number of holes in a binarized image applied to a threshold value, respectively. In other words, *b*_*0*_ stands for the number of white areas surrounded by black backgrounds, corresponding to SPAS. These are measured in continuous binarization processing, which leads to a result without depending on the threshold value. Therefore, *b*_*0*_ quantification based on homology may be superior to or rather supportive of that by simple binarization processing for the comparison of cyst number counts between UHR-CT and NR-CT images. These homology quantification procedures were performed using an in-house homology-based image analysis model. The peak *b*_*0*_ value of homology, which was calculated on each image as a representative of the total count of SPAS, was compared between NR-CT and UHR-CT images using the same linear mixed model as used in the analysis of SPAS counts.

### Analysis 2: Agreement of SPAS size measurement against reference values

The purpose of this analysis was to demonstrate how accurate dimensional measurements of SPAS on CT was with either data acquisition protocol by quantifying the concordance of measurements on original CT images for those on corresponding images of the reference standard images. In this analysis, measurements in original CT images were adopted because discordance of SPAS size measurement in binarized CT images may be overestimated. Smartphone images equivalent to the four phases were used as the reference standard (Fig. 3).

**Fig. 4:**
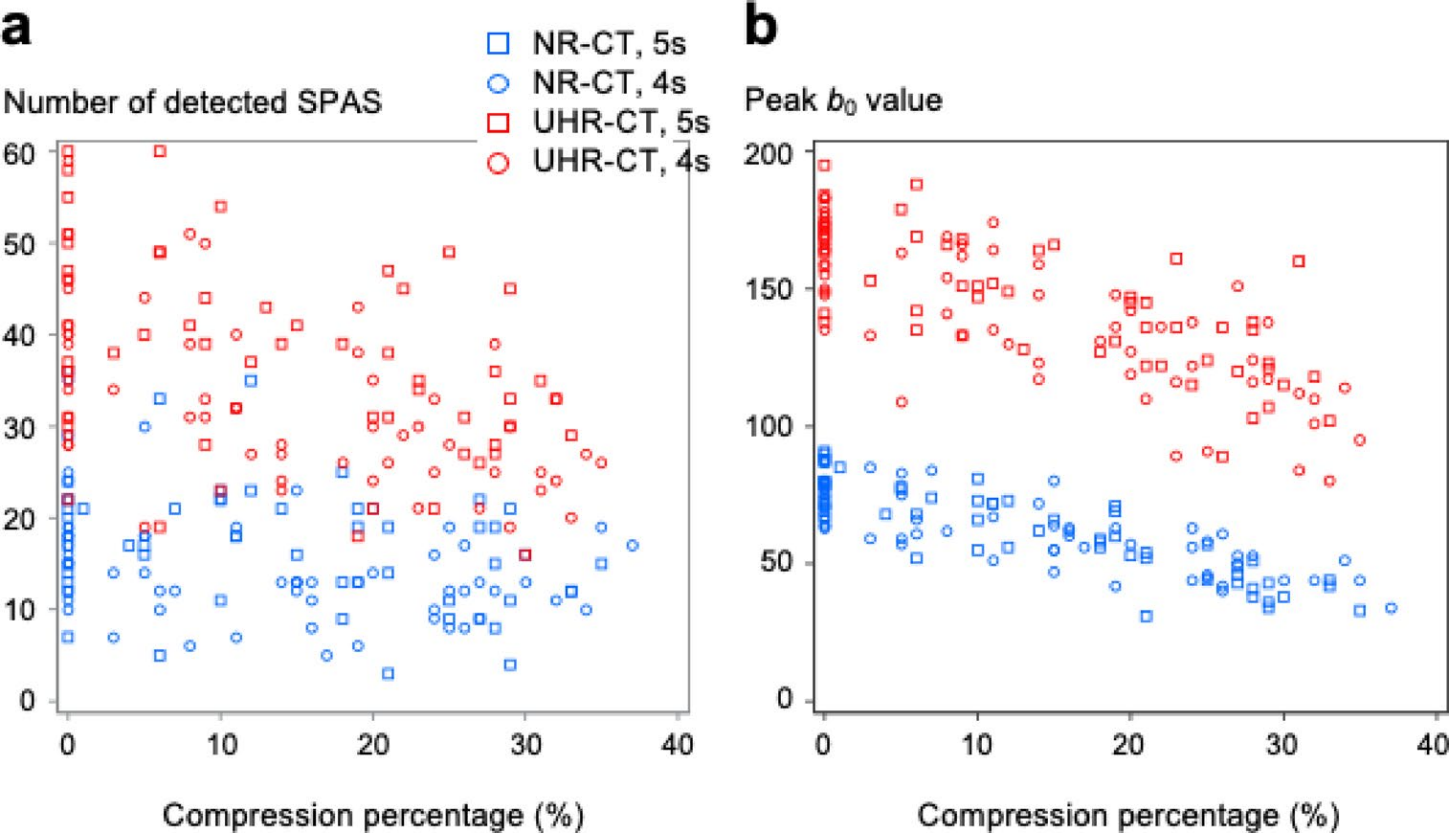
Number of detected simulated peripheral air spaces (SPAS) and peak *b*_*0*_ values according to the compression percentage. Blue circles, blue squares, red circles, and red squares indicate normal-resolution computed tomography (NR-CT) with a 4-s compression cycle (4 s), NR with a 5-s compression cycle (5 s), ultra-high-resolution computed tomography (UHR-CT) with 4-s, and UHR with a 5-s, respectively. (**a**) The scatter plot shows the association between the number of SPAS detected on CT and percentage compression. The slopes of linear regression lines are − 2.0 and − 4.1 for NR-CT and UHR-CT, respectively. (**b**) The scatter plot shows the correlation between the peak* b*_0_ value representing the number of SPAS and percentage compression. The slopes of linear regression lines are − 10.7 and − 17.3 for NR-CT and UHR-CT, respectively.

SPAS visually recognizable on both CT images as well as the smartphone images using either data acquisition protocol were extracted by two radiologists (R.U./radiologist A and A.I./radiologist B) and finally 94 SPAS for each compressing cycle (4 s or 5 s) were selected as measurement targets. The details of these processes are provided in the Supplementary information (S-Fig. 4).

**Fig. 5:**
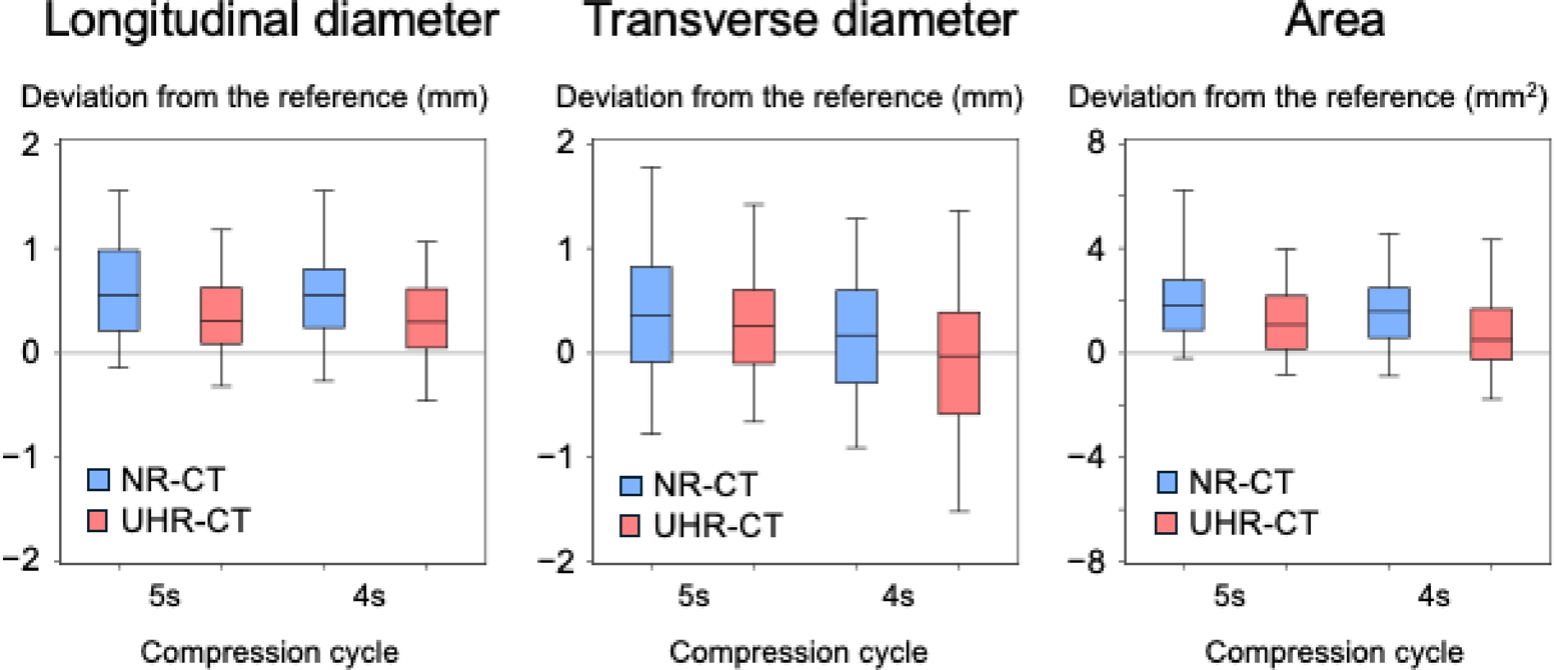
Deviation of the dimensional measurements from the reference for the selected simulated peripheral air spaces (SPAS). Box-and-whisker plots show the deviation for the agreement of the dimensional measurements of 188 extracted SPAS on computed tomography (CT) against the reference standard. The red and blue box-and-whisker plots show results from ultra-high-resolution and normal-resolution CT, respectively. The plots show the 5th, 25th, 50th, 75th, and 95th percentiles.

We defined the moving direction of the plunger of the compressing device as the longitudinal direction, and the orthogonal direction to the moving direction on each of the three cross-sections as the transverse direction. The longitudinal and transverse lines were drawn through the center of each SPAS on original CT images obtained using the two data acquisition protocols, while referring to the drawn longitudinal and transverse lines on original smartphone images (S-Fig. 5a). The profile curve for CT values along with the longitudinal and transverse lines were obtained. For each SPAS, the longitudinal and transverse lengths were obtained based on the full width at half maximum (S-Fig. 5b). SPAS size was calculated as an ellipse based on longitudinal and transverse lengths.

Longitudinal and transverse lines were drawn for each SPAS on binarized smartphone images, and SPAS size was calculated as an ellipse based on longitudinal and transverse lengths.

The deviation of dimensional measurements was calculated by subtracting the value measured in the binarized smartphone image from that measured on CT and compared between NR-CT and UHR-CT images using a linear mixed model. The dependent variable of this model was the deviation of the dimensional measurements of SPAS, and the independent variables were the CT mode (NR or UHR), observer (R.U. or A.I.), position of the cross-section (upper, median, or lateral surface of a sponge phantom), and deviation of the compression percentage between CT and smartphone images (as a continuous variable). This analysis was performed separately for each compression cycle, because different SPAS were selected for each compression cycle.

### Analysis 3: Measurement of the size changes in specific SPAS with an increase in the compression percentage

Continual changes in measured sizes of specific SPAS in association with their compression degree were compared between UHR-CT and NR-CT images by referencing the smartphone images. Radiologist R.U. arbitrarily selected 10 SPAS that were visually recognized on uncompressed cross-sections in UHR-CT and NR-CT images as well as smartphone images.

Continual size changes of the 10 selected SPAS with an increase in the compression percentage were obtained from the longitudinal and transverse diameters of SPAS in each compression phase on smartphone and CT images measured by two radiologists (R.U. and Y.N./radiologist C). These were compared between NR-CT and UHR-CT images using a linear mixed model. The independent variables of this model were the CT mode (NR or UHR), observer (R.U. or Y.N.), position of the cross-section (upper, median, or lateral surface of a sponge phantom), compression cycle (4 s or 5 s), compression percentage (as a continuous variable), and interaction between the CT mode and compression percentage.

All statistical analyses were conducted using SAS 9.4 (SAS Institute Inc., Cary, NC, USA). The significance level was set at *P* < 0.05.

### Additional evaluation using the CT performance phantom

A dedicated phantom (Catphan, TOYO MEDIC CO., LTD, Chiyoda-ku, Tokyo, Japan) was used to investigate the modulation transfer function (MTF), contrast to noise ratio (CNR), and noise magnitudes at various radiation dose levels. MTF at 5% (MTF_5%_) and MTF at 10% (MTF_10%_) as spatial resolution corresponding to the contrast between SPAS and surrounding backgrounds and CNR were measured for a CT density difference of 200 HU. Noise magnitude was measured as the square root of the area under the noise power spectrum curve between 0 and 1 cycle /mm at 120, 80, 40, 20, and 10 mA under other data acquisition parameters set identical as our experimental approach using a simulated sponge phantom. In addition, effective energy was estimated based on aluminium half value thickness using a dedicated semiconductor dosimeter (Accu-Gold+, IBA dosimetry, Schwarzenbruck, Germany).

## Results

### Comparison of counts of SPAS using a simple binarization method (Moment function) between UHR-CT and NR-CT images

Table 1 demonstrates the number of SPAS in each cross-section detected using a simple binarization method in 112 whole cross-sections in each size criteria in both UHR-CT and NR-CT images. The number of SPAS in UHR-CT images with sizes of 0.5 to 1, 1 to 2, and 2 to 4 mm^2^ was approximately 1.5 to 3 times that in NR-CT images, whereas the difference in the number of SPAS between UHR-CT and NR-CT images was low in SPAS of 4–7 mm^2^.

**Table 1:** Number of detected SPAS per image in NR-CT and UHR-CT.

	NR-CT		UHR-CT	
	4-s compression cycle	5-s compression cycle	4-s compression cycle	5-s compression cycle
Total	13.0 (5, 35)	17.5 (3, 36)	30.5 (19, 59)	36.5 (16, 60)
Categorized by SPAS size				
SPAS of 0.5–1 mm^2a^	3.0 (0, 9)	4.5 (1, 12)	12.5 (5, 26)	13.5 (5, 26)
SPAS of 1–2 mm^2a^	4.0 (0, 14)	4.0 (0, 14)	9.5 (3, 25)	11.0 (3, 23)
SPAS of 2–4 mm^2a^	3.0 (1, 10)	5.0 (0, 13)	7.0 (0, 15)	8.0 (1, 20)
SPAS of 4–7 mm^2^	2.0 (0, 8)	3.0 (0, 11)	4.0 (0, 9)	3.0 (0, 9)

Values are presented in median (minimum, maximum). ^a^The range does not include the maximum value.

NR, normal-resolution; SPAS, simulated peripheral air spaces; UHR, ultra-high-resolution.

The number of detected SPAS of 0.5 to 7 mm^2^ decreased as the compression percentage increased, and the slopes seemed to differ between UHR-CT and NR-CT (Fig. 4a). We compared the number of detected SPAS between UHR-CT and NR-CT using a linear mixed model, adjusting for other variables (compression percentage, compression cycle, and cross-section direction). UHR-CT images depicted significantly more SPAS than NR-CT images in the compression percentage between 0 and 35% (S-Table 1), and the slope for the number of detected SPAS against the compression percentage on UHR-CT images was steeper than that on NR-CT images (difference [95% CI], − 2.1 [− 3.7 to − 0.4], *P* = 0.013; S-Table 2). More SPAS were detected in 5-s compression cycles than in 4-s cycles in each cross-section on average (difference [95% CI], 3.9 [1.3 to 6.5], *P* = 0.007), and there were no significant differences in the number of SPAS among the three directional cross-sections (S-Table 2).

**Table 2:** Dimensional measurements of the selected 188 SPAS.

Dimension	Smartphone images (reference)		NR-CT				UHR-CT			
			Radiologist A		Radiologist B		Radiologist A		Radiologist B	
	n	Value	n^a^	Value	n^b^	Value	n^c^	Value	n	Value
5-s compression cycle										
Longitudinal diameter, mm	94	1.3 (0.5, 4.4)	92	1.8 (1.1, 4.6)	94	1.9 (1.1, 4.7)	92	1.7 (0.9, 4.6)	94	1.6 (0.9, 4.1)
Transverse diameter, mm	94	2.7 (1.6, 5.2)	94	3.0 (1.4, 6.2)	93	3.1 (1.9, 5.9)	92	2.9 (1.3, 5.6)	94	3.0 (1.3, 6.6)
Area, mm^2^	94	2.8 (1.1, 11.6)	92	4.4 (1.5, 16.5)	93	4.6 (1.8, 16.7)	92	3.8 (1.1, 18.1)	94	4.0 (1.2, 16.2)
4-s compression cycle										
Longitudinal diameter, mm	94	1.4 (0.5, 2.8)	94	2.0 (1.1, 3.7)	94	2.0 (1.1, 3.6)	94	1.8 (1.0, 3.9)	94	1.8 (0.9, 2.9)
Transverse diameter, mm	94	3.0 (1.6, 5.9)	94	3.0 (1.9, 6.1)	94	3.0 (1.8, 6.2)	94	2.9 (1.4, 6.0)	94	2.8 (1.4, 6.0)
Area, mm^2^	94	3.0 (1.4, 10.6)	94	4.6 (2.4, 12.6)	94	4.6 (1.8, 13.5)	94	3.6 (1.4, 13.2)	94	3.6 (1.2, 12.7)

Values are presented in median (minimum, maximum). ^a^The longitudinal diameter of two SPAS on NR-CT cannot be measured by Radiologist A. ^b^The transverse diameter of one SPAS on NR-CT cannot be measured by Radiologist B. ^c^The diameter of two SPAS on UHR-CT cannot be measured by Radiologist A.

NR, normal-resolution; SPAS, simulated peripheral air spaces; UHR, ultra-high-resolution.

### Comparison of measurement of peak ***b***_***0***_ value representing total count of SPAS using the homology method between UHR-CT and NR-CT images

We measured peak *b*_0_ values representing the total count of SPAS using the homology method as a multi-threshold strategy to support the feasibility of the results obtained by simple binarization depending on the thresholds. The peak *b*_0_ value decreased as the compression percentage increased, and the slopes seemed to differ between UHR-CT and NR-CT (Fig. 4b). We compared peak *b*_0_ values between UHR-CT and NR-CT using the same linear mixed model as for the comparison of SPAS counts. UHR-CT demonstrated significantly larger peak *b*_0_ value than NR-CT images in the compression percentage between 0 and 35% (S-Table 3), and the slope for the peak *b*_0_ value against the compression percentage on UHR-CT images was steeper than that on NR-CT (difference [95% CI], − 6.6 [− 9.4 to − 3.7], *P* < 0.001; S-table 4). A larger peak *b*_0_ value was obtained in 5-s compression cycles than in 4-s cycles in each cross-section on average (difference [95% CI], 4.3 [0.0 to 8.5], *P* = 0.049), and there were no significant differences in peak *b*_0_ value among the three directional cross-sections (S-table 4).

**Table 3:** Dimensional measurements of 10 selected SPAS that were not compressed.

Dimension	Smartphone images (reference)		NR-CT				UHR-CT			
			Radiologist A		Radiologist C		Radiologist A		Radiologist C	
	n	Value	n	Value	n^a^	Value	n	Value	n	Value
Longitudinal diameter, mm	10	1.5 (0.7, 2.2)	10	1.7 (1.5, 3.1)	9	2.1 (1.5, 3.0)	10	1.7 (0.9, 2.2)	10	1.7 (0.9, 4.1)
Transverse diameter, mm	10	2.3 (1.8, 3.2)	10	3.0 (2.4, 4.7)	10	3.1 (2.4, 4.7)	10	3.0 (2.4, 4.6)	10	3.2 (2.4, 4.5)
Area, mm^2^	10	3.0 (1.0, 4.1)	10	4.7 (3.0, 6.7)	9	5.1 (3.0, 8.5)	10	4.0 (1.6, 6.5)	10	5.4 (1.6, 10.1)

Data obtained with the 5-s compression cycle were used for NR-CT and UHR-CT. Values are presented in median (minimum, maximum).

^a^The longitudinal diameter of one SPAS on NR-CT cannot be measured by Radiologist C.

NR, normal-resolution; SPAS, simulated peripheral air spaces; UHR, ultra-high-resolution.

### Agreement of measurement of each SPAS against the corresponding smartphone image as a reference standard

Table 2 summarizes the dimensional measurements of the 188 selected SPAS based on smartphone images, NR-CT, and UHR-CT. We compared the deviation of the dimensional measurements between NR-CT and UHR-CT images using a linear mixed model, adjusting for other variables (observer, cross-section direction, and deviation of compression percentage between CT and smartphone images). The deviation for UHR-CT in the longitudinal diameter was significantly smaller than NR-CT for both the 4-s and 5-s compression cycles (difference in mm [95% CI], − 0.2 [− 0.3 to − 0.1] and − 0.3 [− 0.4 to − 0.2], respectively; both *P* < 0.001; Fig. 5 and S-table 5). In line with this, the deviation in area for UHR-CT was significantly smaller than for NR-CT (difference in mm^2^ [95% CI], − 0.8 [− 1.1 to − 0.5] and − 0.9 [− 1.3 to − 0.6] in 4-s and 5-s cycles, respectively; both *P* < 0.001; Fig. 5 and S-table 5).

### Continual size changes of 10 selected SPAS with increased compression percentage

**Fig. 6:**
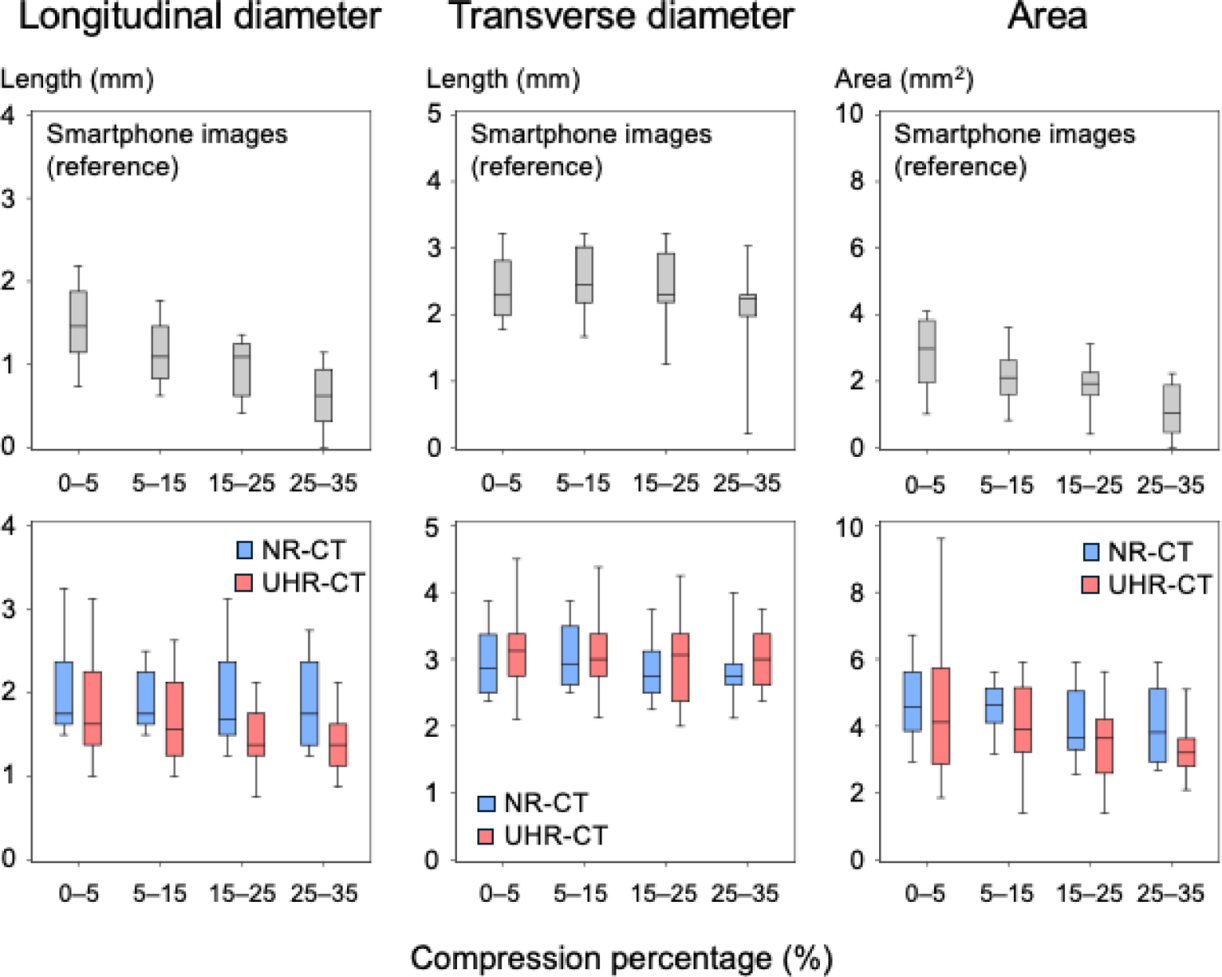
Dimensional measurements for 10 selected SPAS according to the percentage compression. Box-and-whisker plots show continual changes according to the percentage compression in the dimensional measurements of 10 selected SPAS on smartphone images and computed tomography (CT) images in four different compression percentage ranges. The panels in the upper and bottom row represent the measurements on smartphone images and CT images, respectively. The red and blue box-and-whisker plots show measurements from ultra-high-resolution CT and normal resolution CT, respectively. The plots show the 5th, 25th, 50th, 75th, and 95th percentiles.

Table 3 shows the dimensional measurements of the 10 selected SPAS, which were not compressed. For smartphone images, both the longitudinal diameter and area of the 10 selected SPAS decreased as the compression percentage increased (both *P* < 0.001; Fig. 6 and S-table 6). We compared the slopes of continual changes in longitudinal diameter, transverse diameter, and area of the 10 selected SPAS between UHR-CT and NR-CT using a linear mixed model, adjusting for other variables (observer, compression cycle, and cross section direction). The slopes in longitudinal diameter and area of the 10 selected SPAS on NR-CT were not significantly different from 0 (*P* = 0.588 and *P* = 0.146, respectively). In contrast, those on UHR-CT images were all significant (slope for longitudinal diameter in mm [95% CI], − 0.19 [− 0.27 to − 0.11], *P* < 0.001; slope for area in mm^2^ [95% CI], − 0.59 [− 0.87 to − 0.30], *P* < 0.001) for longitudinal diameter and area, respectively (Fig. 6 and S-table 7). The slopes on UHR-CT were steeper than those on NR-CT (difference for longitudinal diameter in mm [95% CI], − 0.16 [− 0.29 to − 0.03], *P* = 0.013; difference for area in mm^2^ [95% CI], − 0.39 [− 0.73 to − 0.05], *P* = 0.023; S-table 7).

### Additional evaluation using the CT performance phantom

The MTF_5%_ for UHR-CT and NR-CT were 1.26 and 0.61 cycles/mm, respectively. The MTF_10%_ for HR-CT and NR-CT were 0.71 and 0.58 cycles/cm, respectively. The CNR for UHR-CT and NR-CT were 13.8 and 13.8, respectively. Noise magnitudes between 0 and 1 cycle /mm were 12.0, 13.3, 14.0, 14.6, and 16.1 for UHR-CT, and 12.4, 13.3, 15.4, 17.8, and 18.9 for NR-CT, at 120, 80, 40, 20 and 10 mA, respectively. Effective energies were estimated as 57.6 and 57.2 keV for UHR-CT and NR-CT, respectively.

## Discussion

This study showed that UHR-CT can more accurately identify dynamic changes for peripheral luminal structures, which is particularly important for dynamic imaging modalities, compared with NR-CT, suggesting that DVCT with UHR mode has significant potential for clinical applications. Quantitative assessment of SPAS in this study revealed that the number of SPAS visualized on UHR-CT images was greater than on NR-CT images, regardless of the compression percentage, and that UHR-CT images provided more accurate size measurements of each SPAS against the corresponding smartphone image used as the reference standard compared with NR-CT images. Additionally, UHR-CT images more effectively depicted changes in SPAS size in proportion to the degree of compression compared with NR-CT images. Based on these results, we believe that DVCT with UHR mode may become a valuable routine examination for assessing respiratory dynamic changes in local lung fields if UHR-CT is further improved in terms of temporal resolution.

The sponge phantoms used in this study were designed to simulate lungs with pathological conditions, such as airway wall thickening or interstitial lung abnormalities, including reticulation or honeycomb formation, rather than normal lungs, because the SPAS sizes in the sponge phantoms were heterogeneous and their walls were thick. In fact, the average CT attenuation values of the sponge phantoms ranged from –816.5 to –729.9 HU. Therefore, the findings of this study can be applied to the evaluation of pathological lungs using DVCT.

Regardless of the compression percentage, the number of SPAS with the size criterion simulating peripheral lung structures depicted on UHR-CT images was greater than on NR-CT images using a simple binarization method. Similarly, the peak *b*_*0*_ values on UHR-CT images were higher than those on NR-CT images when analyzed using homology. The simple binarization method is easy to use; however, its results depend on the threshold values, making the appearance of SPAS in a binarized image similar to that in the original CT image displayed with lung window settings, suggesting that it may be problematic to compare SPAS counts using this method alone. On the other hand, homology is a multi-threshold strategy that was used for the quantitative assessment of emphysema in patients with COPD in a previous study and can provide results representing the total number of isolated pixels without relying on a single threshold value^22^. Therefore, we also applied measurement using homology in this study and found that total presumed number of SPAS depicted on UHR-CT images was greater than on NR-CT images. DVCT with UHR mode may be useful for the quantitative evaluation of air spaces extent separated by interstitial tissue, such as airways, cysts, or emphysema, in static CT as well as DVCT.

In this study, we were able to establish a reference standard using smartphone images, which have much higher spatial and temporal resolution than CT images as a dynamic image modality. The deviation in the measured longitudinal diameter and area relative to the reference standard was smaller on UHR-CT images than on NR-CT images in both compression cycles. This result suggests that the measurement of small air spaces in the lungs on DVCT with UHR mode is superior to using NR-CT images. Establishing a reference standard is usually challenging in studies on dynamic motion imaging, even in animal or experimental studies, whereas histopathological examination is commonly used as a reference standard in static imaging studies. Therefore, this study is more reliable and novel compared with other studies on dynamic imaging.

UHR-CT images can also provide more accurate measurements of respiratory changes in small air spaces. Previous studies have reported that UHR-CT images are desirable in reliable measurements of the size of peripheral air spaces such as peripheral bronchi in the lungs on conventional static CT^2,3^. We confirmed the size of SPAS decreased with an increasing compression percentage by observing smartphone images. UHR-CT images showed gradual reduction in the longitudinal diameters and area of SPAS associated with increasing compression percentages, whereas NR-CT images did not. These results suggest that UHR-CT is essential for measuring respiratory changes in air spaces within the size criterion used in this analysis (around 3 mm^2^: 2 mm diameter).

These results also suggest that UHR-CT images are superior to NR-CT images in depicting peripheral lung structures, in static CT as well as 4D dynamic CT. On the other hand, UHR-CT images are assumed to be more susceptible to motion artifacts with more image noise compared with NR-CT images until now. However, to the best of our knowledge, no study has previously investigated the feasibility of DVCT using UHR mode. In this study, DVCT using UHR mode was demonstrated to be superior for the visualization of peripheral lung structure even in relatively faster lung motion (4-s cycle), which may correspond to abrupt collapse of lung parenchyma. Moreover, application of deep-learning-based reconstruction with DVCT may have contributed to the superiority of UHR mode. We believe that the findings of this study have advanced DVCT to a new stage.

Based on the main results of this study, we believe that DVCT using UHR mode is suitable for evaluating dynamic changes in air spaces with sizes corresponding to airways at the 4th to 5th generation in the upper lung fields (2–4 mm in internal diameter)^2^. Previous studies have shown that volume changes in the upper lungs are generally smaller than those in the lower lungs^23,24^. The mean excursion of the diaphragm during resting tidal breathing is less than 30 mm, whereas the maximum excursion during forced breathing exceeds 80 mm, as reported in other studies using dynamic chest radiography in both patients with COPD and healthy subjects^7,8^. Another study that examined lung tumor motion during resting tidal breathing using 4D-CT for radiotherapy revealed that the maximum motion in three dimensions of a lung tumor in the lower lobe was 26.7 mm^25^. DVCT using UHR mode with a 160-row detector may still be insufficient for evaluating dynamic changes in interstitial lesions such as honeycomb formation in the lower lungs due to its z-axis coverage of only 40 mm even under unforced breathing. Whereas in the upper lung field, DVCT with UHR mode can be sufficiently applied. To analyze dynamic changes in subtle reticulation or regional subpleural heterogeneity in the upper lungs in detail, additional UHR DVCT may be useful, because regional high-resolution CT used to be additionally performed for detailed observation of newly emerged nodular lesions on static CT prior to the clinical introduction of multi-detector CT. Moreover, respiratory-gating CT using a low-pitch spiral acquisition may help overcome the limited z-axis coverage of UHR CT^26,27^, although the inevitably increased radiation dose should be minimized by reducing the tube current with super resolution deep-learning-based imaging reconstruction algorithms.

This study has several limitations. First, we simulated lungs with mild pathological changes, rather than normal lungs, using sponge phantoms, because our focus was on the evaluation of abnormal lung fields. In fact, the mean CT density values of the sponge phantoms were demonstrated to be slightly higher than those of normal lung fields. Therefore, the evaluation of dynamic changes in normal lung fields may require further experiments using phantoms with mean CT density values adjusted to the range of normal lungs. Second, the CT images used for evaluation in this study were not actual surface images of the sponge phantoms, but rather cross-sectional images close to the surfaces, unlike the smartphone images. Additionally, these cross-sections may be slightly distorted during compression, even after adjustments made by the analysis software. Third, image noise might still be counted as SPAS or reflected in the *b*_*0*_ value quantification despite limiting the size criterion on binarized CT images to reduce the likelihood of misrecognizing noise as SPAS. However, noise magnitude quantified using CT performance phantom for UHR-CT was demonstrated to be smaller compared with NR-CT, indicating that image noise did not contribute much to the higher number of detected smaller SPAS for UHR-CT. Fourth, data acquisition in UHR and NR modes was performed on different CT scanners and factors other than spatial resolution, including detector sensitivity, might have influenced the results. Fifth, the tube rotation time was 0.35 s for both UHR-CT and NR-CT, because previous studies on DVCT typically used a tube rotation time of 0.35 s, and the UHR-CT scanner for UHR mode used in this study could not be utilized a 0.275-s rotation time. We matched the imaging conditions between UHR and NR modes because the primary purpose of this study was to compare the spatial resolutions on DVCT. The temporal resolution of DVCT with UHR mode may be further improved in the future if higher tube rotation speeds become available. Sixth, we compared UHR-CT and NR-CT with visually equivalent compression percentages, and in Analysis 2, we used smartphone images as the gold standard with visually equivalent compression percentages. The compression percentages of each triplet consisting of the smartphone, UHR-CT, and NR-CT images are not strictly identical as these images’ appearances were visually matched. However, we included the deviation of compression percentage between CT and smartphone images as an independent variable in the linear mixed model, thereby accounting for these differences in Analysis 2. Seventh, the reproducibility of the SPAS measurements across repeated data acquisitions on NR-CT and UHR-CT and by smartphone was not evaluated, mainly because we focused on assessing whether UHR-CT provides favorable effect on the visualization of dynamic changes in SPAS compared with NR-CT. However, this issue is crucial and should be assessed in another future study with a sufficiently large data set.

In conclusion, we demonstrated that DVCT with UHR mode can provide more detailed information on dynamic changes in peripheral lung structures during unforced breathing compared with NR-CT.

## Supplementary Information

Below is the link to the electronic supplementary material.

Supplementary Material 1

Supplementary Material 2

## Data Availability

The datasets used and/or analyzed during the current study are available from the corresponding author upon reasonable request.
