# Supplementary material for: Investigation into the utility of ultra-high-resolution mode on dynamic-ventilation computed tomography using sponge phantoms
Source: Sci Rep. 2025 Dec 11;16:507. doi: 10.1038/s41598-025-30102-5 (PMC12775385; doi:10.1038/s41598-025-30102-5)
Supplement: Supplementary file 2 — Supplementary Material 2 [file 41598_2025_30102_MOESM2_ESM.docx]

**Title**

Investigation into the utility of ultra-high-resolution mode on dynamic-ventilation computed tomography using sponge phantoms

**Authors**

Ryo Uemura^1^, Yukihiro Nagatani^1^, Jun Matsubayashi^2^, Akitoshi Inoue^1^, Kyohei Iwai^1^, Kenichi Kamiya^3^, Kentaro Doi^4^, Akira Sato^4,5^, Hodaka Numasaki^4^, Kazuaki Nakane^4^, Masahiro Yanagawa^6^, Noriyuki Tomiyama^6^, & Yoshiyuki Watanabe^1^

1 Department of Radiology, Shiga University of Medical Science, Seta Tsukinowa-cho, Otsu, Shiga, Japan 520 2192.

2 Center for Clinical Research and Advanced Medicine, Shiga University of Medical Science, Seta Tsukinowa-cho, Otsu, Shiga, Japan 520 2192.

3 Division of Cardiovascular Surgery, Department of Surgery, Shiga University of Medical Science, Seta Tsukinowa-cho, Otsu, Shiga, Japan 520 2192.

4 Department of Medical Physics and Engineering, The University of Osaka Graduate School of Medicine, 2-2 Yamadaoka, Suita, Osaka, Japan 565 0871.

5 Artificial Intelligence Center for Health and Biomedical Research, National Institutes of Biomedical Innovation, Health and Nutrition, 3‑17 Senrioka‑shin‑machi, Settsu‑shi, Osaka 566‑0002, Japan

6 Department of Radiology, The University of Osaka Graduate School of Medicine, 2-2 Yamadaoka, Suita, Osaka, Japan 565 0871.

**Table of contents**

**Supplementary information with comprehensive methodology**

**(1) Preparation of sponge phantom simulating peripheral air space structures**

**(2) A dedicated compressing device of the sponge phantom simulating ventilation**

**(3) Selection process for evaluation of the measurement concordance using CT images against the standard of reference**

**Supplementary Figure 1. How to determine a simulated sponge phantom**

**Supplementary Figure 2. Selection of an appropriate automatic threshold function**

**Supplementary Figure 3. Measurement of sizes and the total number of simulated peripheral air spaces on binarized CT images**

**Supplementary Figure 4. Selection process for the evaluation of concordance of the measurement using CT images against the standard of reference**

**Supplementary Figure 5. Measurement of longitudinal** **and transverse lengths of a simulating peripheral air space on original CT images**

**Supplementary Table 1. Estimated difference of the number of SPAS between NR-CT and UHR-CT images according to the compression percentage**

**Supplementary Table 2. Results of fitting a linear mixed model to the number of SPAS per image**

**Supplementary Table 3. Estimated difference in the peak *b_0_* value between NR-CT and UHR-CT images according to the compression percentage**

**Supplementary Table 4. Results of fitting a linear mixed model to the peak *b_0_* value per image**

**Supplementary Table 5. Agreement of the SPAS dimensional measurements between CT and smartphone images**

**Supplementary Table 6. Change in dimensional measurements for 10 selected SPAS on smartphone images according to the compression percentage**

**Supplementary Table 7. Change in dimensional measurements for 10 selected SPAS according to the compression percentage**

**Supplementary information for comprehensive methodology**

1. **Preparation of a sponge phantom simulating peripheral air space structures**

Several commercially available sponges made of different materials including polyurethane, cellulose, and melamine were scanned using computed tomography (CT). Image data reconstructed with a slice thickness of 0.5 mm were visually compared with a fixed window level of -650 HU and window width of 1500 HU. Cellulose sponges showed the highest contrast between simulating peripheral air spaces (SPAS) and the sponge matrix and were judged to be the most appropriate material for simulating phantoms that represent peripheral airways or cysts and the surrounding interstitium in the lung. (**S-Fig.1a, b**). However, even in the cellulose sponge, the inside contrast was judged as insufficient for the observation of dynamic image data obtained during continuous scanning. After immersing the sponges in diluted iodine contrast media with a concentration of 15 mg/dl for a day, we squeezed out free contrast media in air spaces and kept the sponges at room temperature for another day to obtain completely dried sponges to amplify the internal contrast (**S-Fig.1c**). Thereafter, the cellulose sponges were cut down into rectangular parallelepiped pieces of approximately 6 × 6 × 3.5 cm, and 8 sponge phantoms were prepared. Finally, after excluding 2 sponge phantoms whose internal structures were revealed to be incompletely dried through the observation of their CT image data, the remaining 6 sponge phantoms were used for the following assessments.

1. **A dedicated compressing device for the sponge phantom simulated ventilation**

A dedicated compressing device based on a conversion mechanism from rotating motion to linear motion was developed to simulate the dynamic change in SPAS during respiration. The device consisted of a plunger, conversion mechanism, and transparent acrylic cube case of about 6.5 × 6.5 × 6.5 cm per side, which can contain a sponge phantom (**Fig.2b**). The plunger powered by electric motors moved like a piston in the transparent case and can compress and decompress a sponge phantom along with its moving direction, to generate simulated respiration (**Fig.2c**). In this study, the maximal compression length on the simulated sponge phantom was fixed as 15 mm. The duration time of the compression cycle was adjustable and set at 4 seconds (compression velocity; about 7.5 mm/s) or 5 seconds (compression velocity; about 6 mm/s).

1. **Selection process for the evaluation of the measurement concordance using CT images against the standard reference**

S-Figure.4 shows an overview of the process and the role of each observer. Images of the smartphone were binarized (**S-Fig.4a**), and excessively small SPAS (less than 2 mm^2^) that were hardly visible on CT images using either data acquisition protocol were excluded using the analyzing particle function with Image J software (**S-Fig.4b**). SPAS for the detection test were extracted considering the size distribution among the remaining SPAS (**S-Fig.4c**).

The following procedures were also performed using Image J software. Scale information was added to smartphone images using the setting scale function. An original smart phone image was first converted into an 8-bit image and then converted into plural binarized ones with another automatic threshold function “auto local threshold function”. The most appropriate function with the highest visual concordance to an original image among binarized ones was determined as Phansalkar. The size of SPAS on binarized images using Phansalkar, from 2 mm^2^, which corresponded to a diameter of approximately 1.6 mm, to infinity in size, were measured using the function for automatically counting particles “Analyze particles” **(S-Fig.3)**, because SPAS with dimensions of less than 2 mm^2^ could not be readily depicted on CT images using either data acquisition protocol.

A radiologist with 25 years’ experience (Y.N./radiologist C) selected 10 SPAS in each 56 (14 × 4) binarized smartphone images and marked corresponding 560 SPAS on the original smartphone images as follows; after all the SPAS detected in each of the binarized images were arranged in ascending order in size, 10 SPAS were selected almost at even intervals in the order with normal distribution in size, by referring to a table showing size and locational information of the SPAS

Two other radiologists with 8 and 14 years of experience (R.U./radiologist A and A.I./radiologist B respectively) determined whether the 560 marked SPAS on original smartphone images were detected on original CT images obtained using each of the 2 data acquisition protocols under the fixed default window level/width (-650 HU/1500 HU in UHR mode and -600 HU/1600 HU in NR mode). Among SPAS that were detected on original CT images obtained using both 2 data acquisition protocols by both radiologist A and B, 94 SPAS were selected by radiologist C, as the selected ones had almost a normal distribution in the size of SPAS, in 56 binarized smartphone images and corresponding 94 SPAS were marked on original smartphone images (**S-Fig. 4e**).

**S-Fig.1.** **How to determine a simulated sponge phantom**

This figure shows an overview of how to determine a simulated sponge phantom

**(a)** A smartphone image of different types of sponges. The dark-blue sponge in a fish-like shape and the pink sponge are made of polyurethane. The white sponge in the middle and the light-blue sponge on the right are made of melamine and cellulose, respectively.

**(b)** A Computed tomography (CT) image of the different types of sponges is shown. The sponge made of cellulose on the right (white arrow) can be recognized; however, the other sponges were hardly visible due to poor contrast with the surrounding air.

**(c)** A CT image of a sponge made of cellulose with iodine contrast immersion on the right and that without on the left is shown. Simulated peripheral air spaces inside the sponge with iodine contrast immersion were more clearly visible thanks to increased contrast between cellulose and internal air.


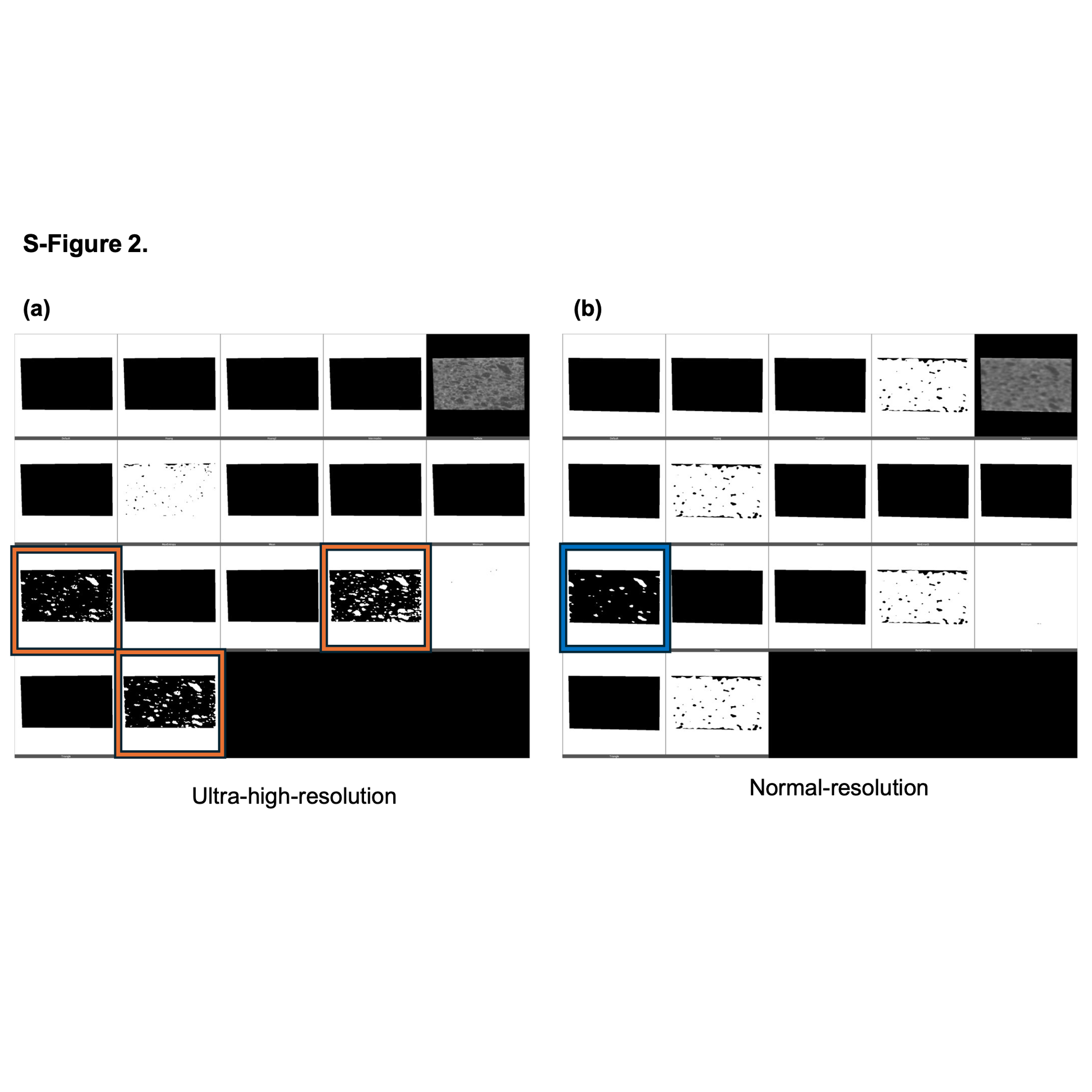


**S-Fig.2. Selection of an appropriate automatic threshold function**

Seventeen binarized images from the automatic threshold function (Auto threshold) on Image J for an original computed tomography (CT) image of a median surface cross-section of the sponge phantom were obtained using ultra-high-resolution (UHR) mode **(a)** and for images obtained using normal-resolution mode (NR) **(b)** are shown on the left and right sides, respectively. Moments, RenyiEntropy, and Yen functions (orange outlines) provide appropriate binarized CT images using UHR mode **(a)**, whereas for CT images obtained using NR mode, binarization was optimal only with the Moment function (blue outline) **(b)**.

**S-Fig.3. Measurement of sizes and the total number of simulated peripheral air spaces (SPAS) on binarized computed tomography (CT) images**

Based on binarized computed tomography (CT) images after conversion into 8-bit images, the sizes and total numbers of SPAS were measured using the function for automatic counting of particles.

**S-Fig.4****.** **Selection process for the evaluation of concordance of measurements using computed tomography (CT) images against the reference standard**

Overview of the selection process for the evaluation of concordance of measurements using CT images against the reference standard and the role of each observer.

**(a)** First, smartphone images were binarized by the Phansalkar.

**(b)** Smaller simulated peripheral air spaces (SPAS) with dimensions less than 2 mm^2^ on binarized smartphone images were excluded using the function for automatic counting of particles by a radiologist (R.U./radiologist A).

**(c)** Second, SPAS for the detection test were extracted considering size distribution among the remaining residual SPAS by another radiologist (Y.N./radiologist C).

**(d)** Third, radiologist A and another radiologist (A.I./radiologist B) checked whether the extracted SPAS could be identified on both original CT images.

**(e)** Fourth, among detectable SPAS on both original CT images (Yellow), 94 SPAS for measurement were selected considering size distribution by radiologist C to be measured in the longitudinal and transverse diameters and area calculated on binarized smartphone images by radiologist C and both original CT images by two radiologists (R.U. and A.I.). Two yellow SPAS surrounded by red outlines stand for selected ones for the measurement of cross-sections.

**S-Fig.5.** **Measurement of longitudinal** **and transverse lengths of simulating peripheral air space (SPAS) on original computed tomography (CT) images**

An example of longitudinal length measurement of a SPAS on ultra-high-resolution CT based on the profile curve of CT density is demonstrated.

**(a)** The white line indicates for the longitudinal dimension drawn by a radiologist (R.U./radiologist A) on a selected SPAS.

**(b)** A profile curve for CT density values on the vertical line obtained using Image J software is shown as a polygonal line graph. The X and Y coordinates of black solid points on the line graph stand for the distance along the white vertical line from the original point at its top end (dark gray arrow in a) and the CT density value, respectively. The dotted horizontal line on the left passes through the half value 1 (-825.5 HU) in a CT density between the first highest peak value (-719.0 HU) at T1 (white arrow in b) and the lowest peak value (-931.9 HU) at B (black arrow in b). Similarly, the dotted horizontal line on the right passes through the half value 2 (-768.3 HU) in the CT density between the second highest peak value (-604.6 HU) at T2 (white arrow in b) and the lowest peak value (-931.9 HU) at B (black arrow in b). A black solid point, which is the nearest to the corresponding one to the half value 1 (-825.5 HU) on the leftward downslope of the polygonal line graph, and had higher CT density than the half value 1 (-825.5 HU) defined as M1 (light gray arrow in b). X and Y coordinates of M1 (light gray arrow in b) were 0.625 and -804.3, respectively. Another black solid point nearest to the corresponding one to the half value 2 (-768.3 HU) on the rightward upslope of the polygonal line graph, and had higher CT density than the half value 2 (-768.3 HU) was defined as M2 (light gray arrow in b). The X and Y coordinates of M2 (light gray arrow in b) were 2.249 and -736.5. In this study, as a matter of convenience, the longitudinal distance was measured as distance between M1 and M2 on the X-axis of the polygonal line graph (1.624 mm = 2.249 – 0.625).

**Supplementary Table 1. Estimated difference in the number of SPAS between NR-CT and UHR-CT images according to the compression percentage**

| **Compression percentage** | **Number of SPAS detected on NR-CT,**  **LS mean [95% CI]** | **Number of SPAS detected on UHR-CT,**  **LS mean [95% CI]** | **Difference,**^a^  **LS mean [95% CI]** | ***P* value** |
| --- | --- | --- | --- | --- |
| 0% (no compression) | 18.5 [14.3, 22.7] | 40.6 [35.9, 45.3] | 22.1 [17.6, 26.6] | <0.001 |
| 5% | 17.5 [13.7, 21.2] | 38.5 [34.5, 42.6] | 21.1 [17.1, 25.1] | <0.001 |
| 15% | 15.4 [12.4, 18.5] | 34.4 [31.5, 37.4] | 19.0 [15.4, 22.5] | <0.001 |
| 25% | 13.4 [10.4, 16.4] | 30.3 [27.8, 32.8] | 16.9 [12.9, 20.9] | <0.001 |
| 35% | 11.4 [7.9, 14.9] | 26.2 [23.3, 29.1] | 14.8 [9.7, 19.8] | <0.001 |

^a^ Difference = UHR-CT – NR-CT.

CI, confidence interval; LS, least square; NR, normal-resolution; SPAS, simulated peripheral air spaces; UHR, ultra-high-resolution.

**Supplementary Table 2. Results of fitting a linear mixed model to the number of SPAS per image**

| **Variable** | **Coefficient [95% CI]** | ***P* value** |
| --- | --- | --- |
| *CT mode* |  |  |
| UHR | 19.2 [15.6, 22.7] | <0.001 |
| NR | Reference |  |
| *Compression cycle* |  |  |
| 5 s | 3.9 [1.3, 6.5] | 0.007 |
| 4 s | Reference |  |
| *Direction of cross-section* |  |  |
| Lateral | 3.3 [−3.0, 9.6] | 0.280 |
| Median | −2.2 [−9.7, 5.3] | 0.538 |
| Upper | Reference |  |
| *Compression percentage*^a^ |  |  |
| For NR-CT, per 10% increase | −2.0 [−3.3, −0.7] | 0.002 |
| For UHR-CT, per 10% increase | −4.1 [−5.6, −2.7] | <0.001 |

^a^ The least square difference (UHR-CT – NR-CT) was −2.1 [95% CI: −3.7 to −0.4] (*P* = 0.013).

CI, confidence interval; NR, normal-resolution; SPAS, simulated peripheral air spaces; UHR, ultra-high-resolution.

**Supplementary Table 3. Estimated difference in the peak *b*_0_ value between NR-CT and UHR-CT images according to the compression percentage**

| **Compression percentage** | **The peak *b*_0_ value for NR-CT,**  **LS mean [95% CI]** | **The peak *b*_0_ value for UHR-CT,**  **LS mean [95% CI]** | **Difference,**^a^  **LS mean [95% CI]** | ***P* value** |
| --- | --- | --- | --- | --- |
| 0% (no compression) | 76.3 [70.9, 81.7] | 166.2 [159.0, 173.4] | 89.9 [80.6, 99.2] | <0.001 |
| 5% | 71.0 [66.0, 75.9] | 157.6 [150.9, 164.3] | 86.6 [78.1, 95.1] | <0.001 |
| 15% | 60.3 [56.0, 64.6] | 140.3 [134.1, 146.5] | 80.0 [72.3, 87.7] | <0.001 |
| 25% | 49.6 [45.3, 53.9] | 123.1 [116.8, 129.4] | 73.5 [65.3, 81.6] | <0.001 |
| 35% | 38.9 [34.1, 43.7] | 105.8 [98.8, 112.9] | 66.9 [57.3, 76.5] | <0.001 |

^a^ Difference = UHR-CT – NR-CT.

CI, confidence interval; LS, least square; NR, normal-resolution; SPAS, simulated peripheral air spaces; UHR, ultra-high-resolution.

**Supplementary Table 4. Results of fitting a linear mixed model to the peak *b*_0_ value per image**

| **Variable** | **Coefficient [95% CI]** | ***P* value** |
| --- | --- | --- |
| *CT mode* |  |  |
| UHR | 80.7 [73.0, 88.4] | <0.001 |
| NR | Reference |  |
| *Compression cycle* |  |  |
| 5 s | 4.3 [0.0, 8.5] | 0.049 |
| 4 s | Reference |  |
| *Direction of cross-section* |  |  |
| Lateral | 3.1 [−7.0, 13.3] | 0.515 |
| Median | 8.1 [−1.5, 17.8] | 0.090 |
| Upper | Reference |  |
| *Compression percentage*^a^ |  |  |
| For NR-CT, per 10% increase | −10.7 [−12.2, −9.2] | <0.001 |
| For UHR-CT, per 10% increase | −17.3 [−19.1, −15.4] | <0.001 |

^a^ The least square difference (UHR-CT – NR-CT) was –6.6 [95% CI: –9.4 to –3.7] (*P* < 0.001).

CI, confidence interval; NR, normal-resolution; UHR, ultra-high-resolution.

**Supplementary Table 5. Agreement of SPAS dimensional measurements between CT and smartphone images**

| **Dimension** | **Deviation of NR-CT,**^a^  **LS mean** **[95% CI]** | **Deviation of UHR-CT,**^b^  **LS mean** **[95% CI]** | **Difference,**^c^  **LS mean** **[95% CI]** | ***P* value** |
| --- | --- | --- | --- | --- |
| 5-s compression cycle |  |  |  |  |
| Longitudinal diameter, mm | 0.6 [0.5, 0.7] | 0.3 [0.2, 0.4] | −0.3 [−0.4, −0.2] | <0.001 |
| Transverse diameter, mm | 0.4 [0.2, 0.5] | 0.3 [0.1, 0.4] | −0.1 [−0.3, 0.0] | 0.083 |
| Area, mm^2^ | 2.0 [1.6, 2.4] | 1.1 [0.8, 1.4] | −0.9 [−1.3, −0.6] | <0.001 |
| 4-s compression cycle |  |  |  |  |
| Longitudinal diameter, mm | 0.5 [0.4, 0.7] | 0.3 [0.2, 0.4] | −0.2 [−0.3, −0.1] | <0.001 |
| Transverse diameter, mm | 0.1 [0.0, 0.3] | −0.1 [−0.2, 0.1] | −0.2 [−0.4, −0.1] | 0.005 |
| Area, mm^2^ | 1.5 [1.2, 1.9] | 0.7 [0.4, 1.1] | −0.8 [−1.1, −0.5] | <0.001 |

^a^ Deviation = NR-CT – Smartphone. ^b^ Deviation = UHR-CT – Smartphone. ^c^ Difference = Deviation of UHR-CT – Deviation of NR-CT.

CI, confidence interval; LS, least square; NR, normal-resolution; SPAS, simulated peripheral air spaces; UHR, ultra-high-resolution.

**Supplementary Table 6. Change in dimensional measurements for 10 selected SPAS on smartphone images according to the compression percentage**

| **Dimension** | **Slope**^a^ **[95% CI]** | ***P* value** |
| --- | --- | --- |
| Longitudinal diameter, mm | −0.27 [−0.34, −0.20] | <0.001 |
| Transverse diameter, mm | −0.10 [−0.21, 0.02] | 0.104 |
| Area, mm^2^ | −0.52 [−0.66, −0.37] | <0.001 |

^a^ The value of the slope represents the change in value per 10% compression.

CI, confidence interval; SPAS, simulated peripheral air spaces.

**Supplementary Table 7. Change in dimensional measurements for 10 selected SPAS according to the compression percentage**

|  | **NR-CT** | | **UHR-CT** | | **Comparison between NR-CT and UHR-CT** | |
| --- | --- | --- | --- | --- | --- | --- |
| **Dimension** | **Slope**^a^ **[95% CI]** | ***P* value** | **Slope**^a^ **[95% CI]** | ***P* value** | **Difference of slopes**^b^ **[95% CI]** | ***P* value** |
| Longitudinal diameter, mm | −0.03 [−0.16, 0.09] | 0.588 | −0.19 [−0.27, −0.11] | <0.001 | −0.16 [−0.29, −0.03] | 0.017 |
| Transverse diameter, mm | −0.08 [−0.15, −0.01] | 0.022 | −0.07 [−0.16, 0.03] | 0.186 | 0.02 [−0.14, 0.17] | 0.843 |
| Area, mm^2^ | −0.19 [−0.46, 0.07] | 0.146 | −0.59 [−0.87, −0.30] | <0.001 | −0.39 [−0.73, −0.05] | 0.023 |

^a^ The value of the slope represents the change in value per 10% compression. ^b^ Difference of slopes = UHR-CT – NR-CT.

CI, confidence interval; NR, normal-resolution; SPAS, simulated peripheral air spaces; UHR, ultra-high-resolution.
